# The health effects of ultrafine particles

**DOI:** 10.1038/s12276-020-0403-3

**Published:** 2020-03-17

**Authors:** Dean E. Schraufnagel

**Affiliations:** Division of Pulmonary, Critical Care, Sleep, and Allergy, Department of Medicine, University of Illinois at Chicago, M/C719, 840 S. Wood St., Chicago, IL 60612 USA

**Keywords:** Translational research, Asthma

## Abstract

Ultrafine particles (PM_0.1_), which are present in the air in large numbers, pose a health risk. They generally enter the body through the lungs but translocate to essentially all organs. Compared to fine particles (PM_2.5_), they cause more pulmonary inflammation and are retained longer in the lung. Their toxicity is increased with smaller size, larger surface area, adsorbed surface material, and the physical characteristics of the particles. Exposure to PM_0.1_ induces cough and worsens asthma. Metal fume fever is a systemic disease of lung inflammation most likely caused by PM_0.1_. The disease is manifested by systemic symptoms hours after exposure to metal fumes, usually through welding. PM_0.1_ cause systemic inflammation, endothelial dysfunction, and coagulation changes that predispose individuals to ischemic cardiovascular disease and hypertension. PM_0.1_ are also linked to diabetes and cancer. PM_0.1_ can travel up the olfactory nerves to the brain and cause cerebral and autonomic dysfunction. Moreover, in utero exposure increases the risk of low birthweight. Although exposure is commonly attributed to traffic exhaust, monitored students in Ghana showed the highest exposures in a home near a trash burning site, in a bedroom with burning coils employed to abate mosquitos, in a home of an adult smoker, and in home kitchens during domestic cooking. The high point-source production and rapid redistribution make incidental exposure common, confound general population studies and are compounded by the lack of global standards and national reporting. The potential for PM_0.1_ to cause harm to health is great, but their precise role in many illnesses is still unknown and calls for more research.

## Air pollution: Nanoparticles linked to disease

Tiny particles found in air pollution enter the body usually through the lungs and disperse to other organs, causing more inflammation and cellular toxicity than larger particles. Dean Schraufnagel from the University of Illinois at Chicago, USA, reviews the way by which nano-sized air pollutants threaten human health. He describes how ultrafine particles measuring less than 100 nanometres in diameter elicit greater inflammatory responses and stay in the lungs longer than larger particles. Repeated contact with extremely small particulate matter can trigger heart disease, diabetes, cancer, neurological disorders and respiratory ailments, especially among children and people with long-term occupational exposure. Much remains to be learned about the disease-causing properties of these nanoparticles and their long-term effects. Further developments in understanding remain handicapped by the lack of international standards and reporting measures.

## Introduction

Air pollution can harm nearly every organ in the body^1,2^, and particulate matter (PM) is the main offender. PM has been classified by particle size, which is an important factor in its health effects. PM_10_ (particles ≤10 µm in diameter), PM_2.5_ (particles ≤2.5 µm in diameter), also called fine particles, and PM_0.1_ (particles ≤0.1 µm in diameter), also called ultrafine particles (this term is used interchangeably with PM_0.1_ in this document), have different health effects that, in part, result from how these particles navigate the small bronchioles and lung defenses. PM_0.1_ are also called nanoparticles because of their size, although many authors restrict the word “nanoparticles” to the 100-nm or smaller particles produced by controlled engineering processes^3^.

Ultrafine particles are dispersed atmospherically in many settings^4^. Examples are found in nature, from forest fires, ocean splashes, and viruses; combustion processes, from vehicular and power plant emissions and tobacco smoking; and synthetic sources, from toner pigment and many engineered products used for microtechnology. These particles may be formed by the coalescence of ions and gaseous molecules produced by combustion, often as acidic and basic ions or other charged species that combine to form more stable molecules or salts. This process, which usually depends on aqueous oxidation, may explain the sulfate levels of London fog of 1952^5^ and the effects of humidity on the symptoms of patients with respiratory disease. Coalescing PM_0.1_ are a major source of PM_2.5_.

The harmful effects of the different PM categories overlap because the corresponding sizes overlap; PM_10_, which include all smaller particles, will have similar effects to those of smaller PMs, although the effects can be distinguished by taking mass into account. PM_10_ and PM_2.5_ are measured by their mass, while PM_0.1_ are measured by particle number.

## Numbers of particles

A typical concentration of PM_0.1_ in ambient air in rural areas is 2610 particles/cm^3^, whereas a roadside concentration may be 48,180/cm^3^, with a mean global concentration of 10,760/cm^3^ ^4^. The large numbers of PM_0.1_ quickly diminish by amalgamation into larger particles and atmospheric dispersion, resulting in local concentrations or “hotspots” near traffic or sites of industrial production. It is paradoxical that PM_0.1_ particle numbers decrease quickly by coalescence, yet remain airborne for extended periods and can travel to other continents. Peak concentrations of traffic emissions occur near curbsides, and these levels are often more than ten-fold higher than the background. The concentrations regress to background by ~500 m from the source^6^. High concentrations are associated with many different conditions, such as high humidity, low air movement, increased number of diesel vehicles, seasons, and traffic acceleration after stopping^3^. Improvements in fuel and engine technology and the use of catalytic converters have reduced the PM mass and carbon monoxide (CO) from automotive exhaust but have increased the number and toxicity of PM_0.1_^7,8^.

Occupational exposures, especially those associated with combustion or high temperature, such as welding or blast furnace operation, may be great. The highest concentrations of PM_0.1_ are found in welding facilities, machine shops, basic metal industries, traffic-related occupations, and restaurants, with concentrations of 0.7–4.7 × 10^6^, 60–450 times higher than the background levels^3^.

As particle sizes decrease, the numbers increase, especially when such particles approach the size of PM_0.1_. Coarse particles (PM_10-2.5_) accounted for <0.1% of the total particle numbers in a study from Shenyang, China^9^. An airborne concentration of 10 µg/m^3^ would require 2.4 × 10^6^ 20-nm particles, but only one 2.5-µm particle/cm^3,10^. With the particle number and total surface area as important parameters, the mass measurements used for PM_10_ and PM_2.5_ are not useful for PM_0.1_. Particle numbers are so much greater for PM_0.1_ that this measurement is used to estimate PM_0.1_ concentrations.

The variation in PM_0.1_ number with season and location was highlighted by a European study that found that Augsburg, Helsinki, and Stockholm had mean annual concentrations of particles between 10,000 and 11,000 cm^−3^, but winter concentrations were almost twice that much (between 10,000 and 20,000 cm^−3^), and summer concentrations were approximately half that much (between 5000 and 6000 particles cm^−3^. Rome and Barcelona, which have greater air pollution, had average annual concentrations of PM_0.1_ of more than 43,000 and 39,000 cm^−3^, respectively, but winter concentrations of 100,000 cm^−3^. This study also reported diurnal and week-day variations, with daily peak levels between 7 a.m. and 10 a.m. in most places. Sunday had ~2/3 of the weekday concentrations^11^. The variation and fluctuating nature of the exposure makes monitoring, gauging their health effects, and setting standards difficult.

The hourly average exposure over a year of PM_0.1_ differs from that of PM_2.5_ in that the distribution of PM_0.1_ is more skewed and widely spread owing to their more rapid reduction and dispersal. The greater diurnal trend for PM_0.1_ results from the variation in vehicular emissions. PM_0.1_ and PM_2.5_ are not well correlated; the ratio of the particle number-to-mass ratio was found to be highest at roadside sites (indicating a prominence of PM_0.1_) and lowest in polluted cities (indicating a prominence of PM_2.5_). Regulating PM_2.5_ may not significantly reduce PM_0.1_^12^.

## Absorption and retention of PM_0.1_

Most inhaled particles of 10 µm or larger in aerodynamic diameter impact the nasopharyngeal membranes. Inhaled particles of 5–10 µm usually land on the airways and are normally removed by alveolar macrophages and lung lymphatics^1^. Particles in the range of 1–2.5 µm usually make their way to the terminal bronchiole, the site of greatest accumulation and tissue destruction, as commonly seen in centrilobular emphysema. Particles <1 µm stay airborne longer and easily gain access to alveoli. Although most PM sizes can be engulfed by cells, PM_0.1_ translocate transcellularly across alveolar epithelial cells by diffusion through the lipid bilayer of the cell walls^13^. It is not just phagocytic cells that pick up material. All cells absorb cellular fragments of senescent, damaged, or normal cells and exchange and recycle molecular material^14^. The cellular fragments (sometimes termed extracellular vesicles) could easily harbor PM_0.1_.

In an in vitro model, investigators showed that positively charged PM_0.1_ penetrated cells 20–40 times more than negatively charged particles^13^. Although this result may be specific to the cells tested, it points out the importance of surface charge. An important property of PM_0.1_ is the large surface-area to mass ratio that allows the particles to carry large amounts of adsorbed materials per unit mass. The large variety of compounds that attach to these particles is likely to be a major cause of their toxicity, but the great variation of adsorbed material makes it more difficult to link PM_0.1_ to specific conditions. Other properties, such as aspect ratio, charge, surface reactivity, solubility, hydrophobicity or polarity, agglomeration state, and the ability to interact with biologic tissue and generate reactive oxygen species, are important determinants of toxicity.

PM_0.1_ that enter alveoli can be retained in surfactant^15^, thus sidestepping the mucociliary escalator clearance mechanisms. The retention half-life of titanium dioxide (TiO_2_) particles of the identical crystalline structure, deposition burden, and conditions in animal lungs was reported to be 170 days for 250-nm particles and 500 days for 20-nm particles^16^. The finer particles caused stronger and more persistent inflammation, with more type II cell proliferation and macrophage impairment and early interstitial fibrotic foci. The small particles also moved into the lung interstitium and periphery more than the larger ones^16^. TiO_2_, which is considered a safe additive to foods, toothpaste, lotions, and many other household products, appears to have many major toxic effects when its particles are in the PM_0.1_ range^17^.

## Health effects

The first interaction site for PM_0.1_ is the lung. The surface area of the lung has been estimated to be more than 100 m^2 18^, but this is commonly estimated by measuring linear intercepts with a 1-µm probe by light microscopy. The estimate of the surface area becomes larger as the sampling probe becomes smaller, accounting for the additional surface area of an irregular surface. The surface area of the lung to a nanoprobe of PM_0.1_ would be orders of magnitude greater than the light microscopy estimates, something Weibel referred to as the “Coast of Wales” effect^19^.

PM_0.1_, along with their toxic baggage, easily reach the large surface of the lung. They subsequently gain access to other organs through the lung vasculature, either through mobile cells or freely in the vasculature and lymph to directly harm distal organs. Another mechanism by which PM_0.1_ cause harm is lung inflammation and the subsequent spread of inflammatory mediators to distal organs. This is considered the main cause of systemic toxicity for larger PM, which are less able to directly access other organs. In addition to having better access, PM_0.1_ have more toxicity in cellular and animal models^20^. For example, low-solubility and low-toxicity PM_0.1_ cause more inflammation in rat lungs than PM_2.5_ of the same material^21^.

Other explanations for the increased toxicity of PM_0.1_ include the fact that many smaller particles may stress alveolar macrophages more than fewer large ones. This could also explain why clearance is dependent on particle size. Inflammation in response to noxious material on the surface of particles and interactions with the cell surfaces of host tissues would also be greater with ultrafine particles^22^. Another important point when considering human population studies is that PM_2.5_ have more immediate effects, while PM_0.1_ have more delayed effects and a greater influence on mortality^23^. Most epidemiologic studies of acute exposure to PM_0.1_ take into account a lag time between exposure and symptoms of 1–5 days to account for the delayed effect.

Compared to data on PM_2.5_ and PM_10_, there is a paucity of information on the long-term health effects of PM_0.1_. A major reason for this is the lack of international standards and national reporting. Although PM_0.1_ can be readily measured in the atmosphere, the measuring instruments are not standardized, which means methods and protocols vary^24^. Developing standards is difficult for populations because of the variable nature of personal exposures and the silent nature of the effects of air pollution, particularly PM_0.1_. Neither clinicians nor the public generally consider air pollution as a cause of a specific illness. Many exposures, such as to office printers, would go unnoticed by most workers.

The study of PM_0.1_ has been aided by engineered nanoparticles. Ultrafine particles can be manufactured with a high degree of accuracy with regard to their size, shape, and composition. The biological effects of these factors can then be studied more precisely. Engineered nanoparticles can be applied to cells and tissues and given as a challenge to animals and human volunteers. For example, high doses of fibrous and tubular nanostructures can result in fibrotic lung reactions and an increased risk of carcinogenesis^25^. Single-walled nanotubes can persist deep in the lung and induce inflammatory and fibrotic reactions^26^. Many other studies have shown various intracellular effects depending on the species studied and the nature of the nanoparticle^25^.

## Diseases

A review of all diseases associated with PM_0.1_ is beyond the scope of this paper, but the following discussion highlights major associations. Air pollution and its effects on different organ systems have recently been reviewed^1,2^.

### Respiratory

The respiratory system is usually the first line of entry into the body for air pollution, but ingested PM_0.1_ pass through the gastrointestinal tract and can stimulate immune responses in animals and human colonic biopsies. In a Western diet, more than 10^12^ ultrafine particles are ingested daily by a single person^27^. Intact skin is generally considered protective. Short-term exposure to PM_0.1_ with a high content of polycyclic aromatic hydrocarbons increases 8-hydroxyl-2-deoxyguanosine, a byproduct of DNA oxidative damage, in children with eczema but not in those without eczema^28^, although particles of zinc and TiO used in topical skin care have been shown to penetrate intact skin, especially in watery or oily vehicles^29^.

### Metal fume fever and polymer fume fever

Metal fume fever is an example of a disease most likely caused by PM_0.1_. PM_0.1_ are produced by welding. Inhaling the small particles generates a great amount of reactive oxygen species, leading to inflammation^30^. Metal fume fever usually presents with malaise, fever, chills, arthralgias, and myalgias 4–8 h after exposure to metal fumes, usually through welding. Chest radiographs are inconsistent, and the syndrome generally abates without treatment. Although zinc oxide (ZnO) has been incriminated, other metals, namely, copper, magnesium, and cadmium, have also been identified. A similar syndrome, polymer fume fever, has been associated with inhalation of fluorinated polymer products, such as polytetrafluoroethylene (Teflon^®^). Heated polytetrafluoroethylene contains ultrafine particles (median diameter 26 nm) that are toxic to rats, causing hemorrhagic pulmonary inflammation and death with high-dose exposure and decreased function with low-dose exposure^31^. Short-term, high-level exposure to PM_0.1_, such as through diesel exhaust, also causes lung inflammation^32^.

The respiratory mucociliary apparatus is a major tool to clear inhaled particles. Although ultrafine particles can be trapped in the mucous layer, its role in clearance is far less than that for PM of greater density, which impacts the airway. Many lung conditions impair mucociliary function. Ciliary dysfunction is common in smokers and those with respiratory tract infections and may account for the greater vulnerability of these individuals to air pollution. Bronchospasm and cough, which are part of asthma, are common reactions to inhaled irritants, which may account for the increased sensitivity in persons with asthma.

Particulate air pollution is a well-known cause of exacerbations and mortality in persons with chronic obstructive pulmonary disease (COPD), but the role of PM_0.1_ is unclear. A study from Scotland did not find PM_0.1_ to be more harmful than PM_10_^33^, but other studies have reported that indoor biological PM_0.1_ in the form of bacterial extracellular vesicles do cause inflammation and emphysema^34^.

Ambient exposure to PM_0.1_ is associated with cough, reduced peak expiratory flow^35^, and the increased use of medicines^36^ and hospital admissions for persons with asthma^37^. Clinical visits for respiratory illness are associated with increased levels of PM_0.1_^38^. Although most studies have found an increase in asthma symptoms, a study of more than a million adult residents of Toronto did not find evidence for an association between long-term exposure to PM_0.1_ and respiratory disease after adjusting for PM_2.5_, NO_2_, and other covariates^39^. An Australian controlled study also did not find particle number to be independently associated with respiratory symptoms, asthma diagnosis, or lung function, although PM_0.1_ was associated with an increase in inflammatory markers in atopic participants^40^.

### Cardiovascular

Many studies have shown that PM causes systemic inflammation and coagulation changes predisposing to ischemic cardiovascular disease, as measured by elevated C-reactive protein (CRP), circulating polymorphonuclear leukocytes, platelets, fibrinogen, plasma viscosity and other markers. PM promotes endothelial dysfunction, vascular inflammation, and atherosclerosis^1^. Past studies have attributed this effect mainly to PM_2.5_, but a growing body of literature shows that PM_0.1_ have a major role in essentially all of these factors^41-43^. In fact, most studies show a far greater effect for PM_0.1_. PM_0.1_ also cause increased heart rate variability, loss of sympathovagal balance, and altered inflammatory and hemostatic function in exposed humans^44^.

Even brief exposures to PM_0.1_ can cause cardiac effects. In middle-aged individuals with metabolic syndrome, exposure to PM_0.1_ for 2 h caused electrocardiographic changes, a decrease in blood plasminogen and thrombomodulin and an increase in CRP and serum amyloid A^45^.

Many studies have shown an association between chronic exposure to PM_0.1_ and heart disease. A prospective study of 33,831 Dutch residents found that long-term exposure to PM_0.1_ (measured by land use regression) was associated with an increased risk for cardiovascular disease, myocardial infarction, and heart failure^46^. In adults living in Toronto from 1996 to 2012, an increase in PM_0.1_ exposure was associated with an increased incidence of heart failure and acute myocardial infarction. Adjustment for PM_2.5_ and NO_2_ did not change these associations, although NO_2_ was also independently associated with increased heart failure incidence^47^. Mobile neighborhood monitoring found the annual average particle number exposures to be associated with stroke, ischemic heart disease, and hypertension^48^. Other studies have also found increased ischemic and thrombotic stroke with PM_0.1_ exposure^49^ and increased blood pressure and worse microvascular function with PM_0.1_ but not with PM_2.5_ and PM_10_^43,50^.

Particle size has been correlated with total and cardiovascular mortality, with the correlation becoming stronger as the particle size decreases. PM <0.50 µm had the highest correlation^9,51^. No association was found for mass concentrations (PM_2.5_ and larger)^51^.

Particle numbers are associated with cardiovascular disease-related emergency department visits, with a lag of 4–10 days; 10–50 nm particles mainly account for this finding. PM_0.1_ were reported to account for more than 7% of emergency department visits^52^. The strongest correlate of immediate effect (within 2 days) was found with 30–100-nm particles, despite a small mass concentration. The immediate effect related to mass concentration was with the 1–5 µm particles, which had a similar delayed effect to the PM_0.1_ number^52^.

A study of more than 100,000 women in California found that mortality from ischemic heart disease was more strongly associated with PM_0.1_ than with PM_2.5_^53^. Repeated biweekly submaximal exercise tests on adult subjects with stable coronary heart disease showed that PM_0.1_ were associated with electrocardiographic ST-segment depression of >0.1 mV. The researchers found that the PM_0.1_ effect was independent of PM_2.5_. NO_2_ and CO were also associated with a risk for ST-segment depression, but coarse particles (PM_10–2.5_) were not^54^.

However, a study from Denmark of 6515 airport workers who were exposed to long-term PM_0.1_ found no correlation with ischemic heart disease or cerebrovascular disease compared to the corresponding measures in a similar group of unskilled workers^55^.

### Central nervous system

Many articles have been published on the brain or neural effects of PM, and there is a great deal of animal work on the mechanisms by which PM_0.1_ affect the brain and its development. Translocated PM_0.1_ can be found in the brain within 4–24 h after inhalation. Nasal PM_0.1_ can travel up the olfactory nerves to the brain. Animals exposed to aerosols of PM_0.1_ have the greatest brain uptake in the olfactory bulb, even 7 days after exposure. In an animal inhalation study, up to 20% of the PM_0.1_ deposited on the olfactory mucosa moved to the olfactory bulb^56^. This pathway, which could circumvent the blood brain barrier, may be even more direct in humans^57^. PM_0.1_ not only translocate and directly damage neural tissue but also affect autonomic function. Exposure to PM_0.1_ increases sympathetic nervous system activity by decreasing norepinephrine clearance, a feature that is increased with concurrent ozone (O_3_) exposure^58^.

Considerable animal research has been carried out on the effect of PM_0.1_ on brain development. Postnatally, PM_0.1_-exposed animals show short-term memory impairment, cortical and hippocampal changes, which raise the potential for excitotoxicity, and long-term glial activation, which is associated with a wide range of behavioral and other neurologic effects^59^. Pregnant mice administered carbon black nanoparticles intranasally gave birth to offspring with a dose-dependent, long-term activation of astrocytes. Many mRNA level changes associated with angiogenesis, cell migration, proliferation, chemotaxis, and growth factors caused the authors to speculate that this exposure could have wide-ranging implications for health in later life^60^. Other animal studies have shown that PM_0.1_ affects emotional behavior, learning capability, neurotransmission, spontaneous motor activity, and avoidance of performance^61^.

In humans, regular exercise has been shown to improve brain cognition and memory. One mechanism for this effect may be by upregulating brain-derived neurotrophic factor (BDNF). BDNF was measured in subjects before and after cycling for ~20 min near major traffic and, on another occasion, in a room with air filtered to remove PM_0.1_ as well larger PM. The average PM_0.1_ was 28,180 particles/cm^3^ near the road and 496 particles/cm^3^ in the air-filtered room. Serum BDNF concentration rose after cycling in the air-filtered room but not after cycling near the major traffic route^62^.

### Children

Children are more vulnerable to the health effects of air pollution, and these effects may begin with in utero exposure and have lifelong consequences^1^. Exposure of pregnant women to PM_0.1_ increases the risk of low birthweight, especially in those living within 50 m of heavy traffic^63^.

A study from Ghana monitored the personal exposure of 61 junior high school students 24 h per day for 10 weeks. The exposures varied greatly depending on place of residence and the type of activities in which the students were engaged. The highest exposures were in a home near a trash burning site, in a bedroom with burning coils employed to abate mosquitos, in a home with an adult smoker, and in home kitchens during domestic cooking^64^. A similar study conducted in the Pearl River delta in China that monitored school children (aged 9–13) for 2 weeks found that the highest exposures were indoors, associated with smoking adults and the use of mosquito repellent incense^65^. These studies show that the sources of PM_0.1_ are not always predictable and are not only related to vehicular traffic. Microenvironments and chance exposure have important implications for epidemiologic studies.

A major source of PM_0.1_ for children living in rural areas can be exhaust from school buses, especially if they are older diesel-powered vehicles. Filtration of the inside air in school buses has been shown to significantly decrease the number of in-cabin ultrafine particles^66^. Exposure also occurs when children are in the playground and standing outdoors while waiting to enter the bus. Idling engines produce more PM_0.1_ than driving does^67^. These exposures can be reduced by anti-idling policies.

Electronic cigarettes are now commonly used by children and are a source of inhaled PM_0.1_^68^. Electronic cigarettes use heated vehicles (usually propylene glycol and glycerol) to deliver microaerosols to the lungs. They also deliver PM_0.1_ to the brain along with nicotine. High-resistance coils in electronic cigarettes and increased glycerol generate larger particles, and higher coil temperatures generate smaller particles. Particles produced by the electronic cigarettes are deposited in alveoli^69^.

### Diabetes

Air pollution affects many metabolic functions and has been associated with diabetes and other metabolic illnesses. Exposure to PM_0.1_ and NO_2_ from traffic-related sources increases the risk for hypertension and diabetes^70^. A single 2-h inhalation of elemental carbon PM_0.1_ had an effect on heart variability in diabetic subjects that lasted for hours^71^. PM_0.1_ cause autonomic dysfunction^72^ and affect glucose tolerance in patients with diabetes^73^.

### Cancer

Many studies in animals and human cells have shown that PM is mutagenic and tumorigenic^61^, and it appears that the finer the particle size, the greater the mutagenic potential^74^. PM_0.1_ from the atmosphere and roadside contain many mutagenic particles^75^. PM_0.1_ have been shown to produce tumors in rats. The strongest tumorigenic factor was the total surface area of the retained particles, although the dose, particle type, and duration of exposure were also important. Smaller aggregated ultrafine TiO_2_-induced lung tumors in rats much more than larger sized TiO_2_^22^. The carcinogenic properties of nanoparticles are related to their aspect ratio and rigidness^76^.

## Conclusions

The potential for PM_0.1_ to cause harm to health is great, but their precise role in many illnesses is still unknown. Their high point-source production and rapid redistribution make incidental exposure common for the general population and confound general population studies. This has, no doubt, contributed to the lack of global standards and national reporting. The absence of standards and reporting may account for the dearth of standardized measurements, instruments, and protocols. Air pollution is a silent epidemic^77^, and PM_0.1_ may be the quietest of the pollutants. PM_0.1_ vary greatly with the toxins they adsorb, adding complexity to public research. In many ways, the study of PM_0.1_ is at the frontier of air pollution research. As they are better understood, these particles should be more easily controlled. PM_0.1_, as with other air pollution, are avoidable and correctable health risks. Halting or reducing pollution should promptly result in improved health status^78^. Undoubtedly, more research is needed.
